# Active transport of cytoophidia in *Schizosaccharomyces pombe*

**DOI:** 10.1096/fj.201800045RR

**Published:** 2018-05-21

**Authors:** Hui Li, Fangfu Ye, Jing-Yi Ren, Peng-Ye Wang, Li-Lin Du, Ji-Long Liu

**Affiliations:** * Department of Physiology, Anatomy, and Genetics, Medical Research Council Functional Genomics Unit, University of Oxford, Oxford, United Kingdom;; † Key Laboratory of Soft Matter Physics, Beijing National Laboratory for Condensed Matter Physics, Institute of Physics, Chinese Academy of Sciences, Beijing, China;; ‡ School of Physical Sciences, University of Chinese Academy of Sciences, Beijing, China;; § National Institute of Biological Sciences, Beijing, China; and; ¶ School of Life Science and Technology, ShanghaiTech University, Shanghai, China

**Keywords:** cytoophidium, actin filaments, motor proteins, microtubules

## Abstract

The metabolic enzyme cytidine triphosphate synthase has recently been found to form micrometer-sized filamentous structures termed cytoophidia, which are evolutionarily conserved across prokaryotes and eukaryotes. The cytoophidium represents a novel type of membraneless organelle and behaves dynamically inside the cell. The question of how cytoophidia transport is mediated, however, remains unanswered. For the first time, we detected in this study the active transport of cytoophidia, taking advantage of the fission yeast *Schizosaccharomyces pombe* as an excellent model for studying membraneless organelles. We demonstrated that actin filaments, not microtubules, are responsible for this transport. Furthermore, we determined that Myo52, a type of myosin V, is required for the active transport of cytoophidia. These results reveal the major players critical to the dynamics of cytoophidia and extend our understanding of intracellular transport of membraneless organelles.—Li, H., Ye, F., Ren, J.-Y., Wang, P.-Y., Du, L.-L., Liu, J.-L. Active transport of cytoophidia in *Schizosaccharomyces pombe*.

Compartmentation contributes to the heterogeneity and complexity of cellular organization, which are vital to cell survival (1). Thus, it is not surprising that intracellular compartments such as membrane-bound organelles have been one of the core topics in cell biology in the past century. The cell depicted in many classic cell biology textbooks usually contains a handful of membrane-bound organelles floating in a homogeneous soup of cytosol. However, this view has been challenged in recent years. Growing studies have documented that compartmentation can be achieved *via* the formation of large-scale aggregates without membranes (2–5). For example, many membraneless organelles such as cytoplasmic processing bodies (6), histone locus bodies (7), uridine-rich small nuclear RNP bodies (8), and purinosomes (9) have been identified inside the cell.

In 2010, 3 groups reported that cytidine triphosphate synthase (CTPS) can form filamentous structures termed cytoophidia in bacteria, budding yeast, and *Drosophila* (10–12). Subsequently, CTPS–containing cytoophidia have been found in human cells (13, 14). Thus, the cytoophidium represents a novel type of membraneless organelle, which is evolutionarily conserved across prokaryotes and eukaryotes (15, 16).

Intracellular transport and dynamics of organelles are essential to many cellular functions. For example, the Golgi apparatus is generally trafficked to the perinuclear area, melanosomes move from the central region of the cell to the cell periphery in response to hormonal stimuli, and mitochondria are transported in the axon and dendrites of a neuron to regulate synaptic homeostasis (17, 18). It is well known that transport of most membrane-bound organelles are driven along microtubules and microfilaments. In a recent study, we found that CTPS can form filamentous cytoophidia in the fission yeast *Schizosaccharomyces pombe* (19). We have also observed that cytoophidia can move around inside the yeast cells. However, how the cytoskeleton contributes to cytoophidium movement remains elusive.

To understand the dynamics of membraneless organelles, here we use the cytoophidia in single living *S. pombe* cells as an example. We sought to answer 3 key questions: *1*) Do cytoophidia undergo active transport? *2*) Which cytoskeleton is responsible for the dynamic behavior of cytoophidia? *3*) Which motor protein drives cytoophidium transport?

## MATERIALS AND METHODS

### *S. pombe* strains

An *S. pombe* strain expressing CTPS-yellow fluorescent protein (YFP) from the endogenous promoter at the endogenous locus was generated as described before (19). The Lifeact-GFP strain was obtained from Mohan Balasubramanian (University of Warwick, Coventry, United Kingdom). The *Myo52* gene was deleted from the CTPS-YFP expressing strain using PCR-based gene targeting and the deletion was verified by colony PCR (20, 21).

### *S. pombe* culture and drug treatment

All the cells were cultured in yeast extract at 32°C with 3 supplements: adenine, leucine, and uracil. The cells are at log phase below 1.0 optical density at 600 nm (OD_600_). Only the cells with a 0.3–0.7 OD_600_ value were used for the experiments. To disrupt the microtubules or actin filaments, the cells were incubated for 30 min with 25 µg/ml carbendazim (MBC; MilliporeSigma, Burlington, MA, USA) or 10 µM latrunculin A (LatA; Abcam, Cambridge, United Kingdom), respectively. To inhibit Myo52, the cells were incubated for 30 min with 2 µM pentabromopseudilin (PBP; AdipoGen, San Diego, CA, USA). For all the control experiments, the cells were treated with 1% DMSO.

### Live cell fluorescence imaging

For the imaging, the *S. pombe* cells were placed in glass-bottomed Petri dishes (MatTek, Ashland, MA, USA) and then covered with agarose gel pads containing adenine, leucine, and uracil to immobilize the cells. The cytoophidia containing CTPS-YFP in live cells were imaged under 2 systems: Nikon A1R confocal microscopy and single-molecule fluorescence microscopy (Tokyo, Japan). The confocal microscopy is equipped with a ×60 water-immersed objective [numerical aperture (NA) 1.2] and an incubation chamber (Solent Scientific, Fareham, United Kingdom). The final pixel size is 0.21 µm. The single-molecule fluorescence microscopy is built from Olympus ×70 microscopy and equipped with an incubation system (Tokai Hit, Shizuoka, Japan). Lasers at different wavelengths are combined and focused at the back focal plane of the ×60 oil-immersed objective (NA1.4). Wide-field and highly inclined and laminated optical sheets were used for the imaging. The emission signal was collected by an EMCCD (Ultra897; Oxford Instruments, Abingdon, United Kingdom). The corresponding pixel size was 0.267 µm. Under both systems, all live cell imaging was performed at 32°C, a laser at 488 nm was used to excite YFP, and the emission was collected through a 525/50 nm filter. Images were acquired every 0.258 s for 5 min on a fixed focal plane. The time resolution was sufficient to follow the movement of cytoophidia. In the case of imaging Lifeact-GFP, the cells were imaged by confocal microscopy.

### Particle tracking

Tracking of cytoophidia was accomplished using the ImageJ plugin Particle Tracker (22). The raw footage was first partitioned into separate clips containing 1 cell each. Only the cells in interphase were selected for cytoophidium tracking. For each frame, only cytoplasmic cytoophidia were detected and localized under the parameters: a radius of 5 pixels and a percentile of 0.2% (meaning the top 0.2% bright pixels were considered for candidate cytoophidia). Under this setting, the small cytoophidia in the nucleus were easily excluded due to their lower intensity, as were the out-of-focus cytoophidia in the cytoplasm. The linking range and displacement were both set to 3, sufficient to link the cytoophidia between frames. Because there is typically only 1 cytoplasmic cytoophidium in each cell, incorrect linking between different cytoophidia is rare. All the detecting and linking steps were optically confirmed. The effectiveness of the software for tracking cytoophidia was verified by imaging the immobilized cytoophidia in fixed cells under the same condition. The localization accuracy of the system was ∼35.8 nm, uninfluenced by any variation in cytoophidia length. Only the trajectories of cytoophidia longer than 118 frames (about 30 s) were selected and saved in the database for further analysis.

### Data analysis

User-defined Matlab programs were employed for data analysis. For each trajectory, according to the plot of *x*(*t*) or *y*(*t*), the segments in unidirectional displacements were manually selected and saved as separate .txt files. Then the speed and run length for each segment were calculated. To prevent the influence of rotation of the cytoophidia and random fluctuation, only the segments with speed >0.05 µm/s and run length >1 µm were chosen as directed phases. The MSD curve for each trajectory was calculated according to the equation MSD(τ) = |*r*(*t* + τ)−*r*(*t*)|^2^, where τ is the time lag. The diffusion rate of the trajectory was determined by linear fitting the first 10 points with MSD(τ) = 4*D*τ + *c*. To study the transit directed motion in the trajectories, a local MSD analysis was applied: for each analysis, a series of 40 consecutive points along a trajectory were selected and used to calculate the local MSD, which was then fitted by the power law MSD_local_(τ) ∼ τ^α′^. The temporal exponent α′ indicates the nonlinear relation between MSD_local_ and time, and contains information about local motion modes. In contrast to the MSD of the whole trajectories, the MSD_local_ and the corresponding temporal α′ fluctuate in a wide range because the samples were limited to 40 points. For free Brownian motion, α′ centers around 1, with a distribution ranging from 0.5–1.5. For diffusion within a confined area, the distribution of α′ shifts toward 0, with the center below 1. For diffusion overlaid with directed motion, the distribution of α′ shifts toward 2, and the upper limit can be >1.5, so the percentage of α′ >1.5 represents active motion. Data are presented as means ± sem.

## RESULTS

### Active transport of cytoophidia in *S. pombe*

**Figure 1:**
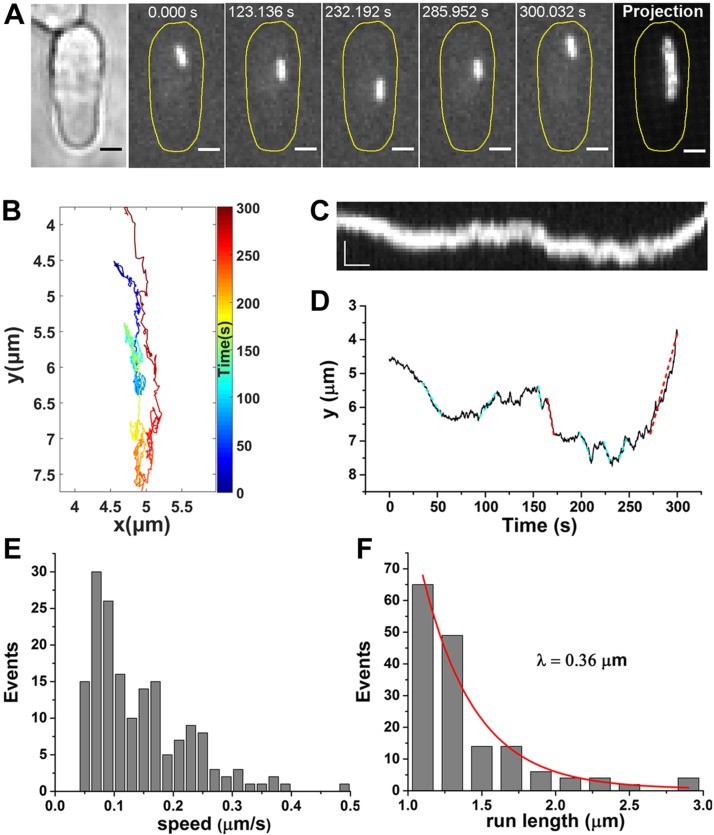
Cytoophidia undergo active transport in *S. pombe*. *A*) Sample images of time series show 1 cytoplasmic cytoophidium moving back and forth in the *S. pombe* cell. Control cells were treated with 1% DMSO. CTPS was fused to YFP and expressed under the endogenous promoter. Far right, the maximum projection of the clip. Scale bars, 2 µm. *B*) Trajectory of the cytoophidium with colored segments corresponding to the time points. *C*) Kymograph of the cytoophidium. Scale bars, 2 µm (vertical) and 20 s (horizontal). *D*) Position of cytoophidium *vs*. time elapsed. The discernable directed displacements are marked with dashed lines. The red dashed lines indicate the valid directed phases with the speed >0.5 µm/s and run length >1 µm. *E*) The speed distribution of the directed phases of cytoophidia. *F*) The run-length distribution of the directed phases of cytoophidia. The red curve shows the exponential fit *y* = *e*^−^*^x^*^/λ^, yielding the run length λ. There were 175 directed phases.

We performed live cell imaging and single-particle tracking of the cytoplasmic cytoophidia of which CTPS was fused to YFP (19). The micrometer-size filamentous structures diffused throughout the whole *S. pombe* cells. The tracking method we used is effective and accurate, and only the movement of the centroid of cytoophidia was analyzed (Supplemental Fig. S1). We observed that a cytoophidium moved back and forth along the long axis of the *S. pombe* cell (**Fig. 1*A–C*** and Supplemental Movie S1). We further quantified cytoophidium movement by analyzing the consecutive points of *y*(*t*) (Fig. 1*D*). The trajectory reveals several periods of rapid, unidirectional displacement marked by dashed lines interspersed with periods of nondirectional motion. Considering the length of filamentous cytoophidia in *S. pombe* (∼2 μm), we identified any unidirectional displacements longer than 1 μm as directed phases (red dashed lines). The short displacements (cyan dashed lines) were excluded to avoid false detection that could result from the rotation of the cytoophidium. By analyzing 202 cytoophidium trajectories in *S. pombe* cells, 175 directed phases were detected, allowing us to characterize the active motion of individual cytoophidia. We found that the speed ranged from 0.05–0.5 μm/s, with a mean speed of 0.15 μm/s (Fig. 1*E*), and that the mean run length (λ) was 0.36 μm (Fig. 1*F*). The unidirectional movements clearly indicate that cytoophidia undergo active transport in *S. pombe* cells.

### Microtubules are not involved in the active transport of cytoophidia

**Figure 2:**
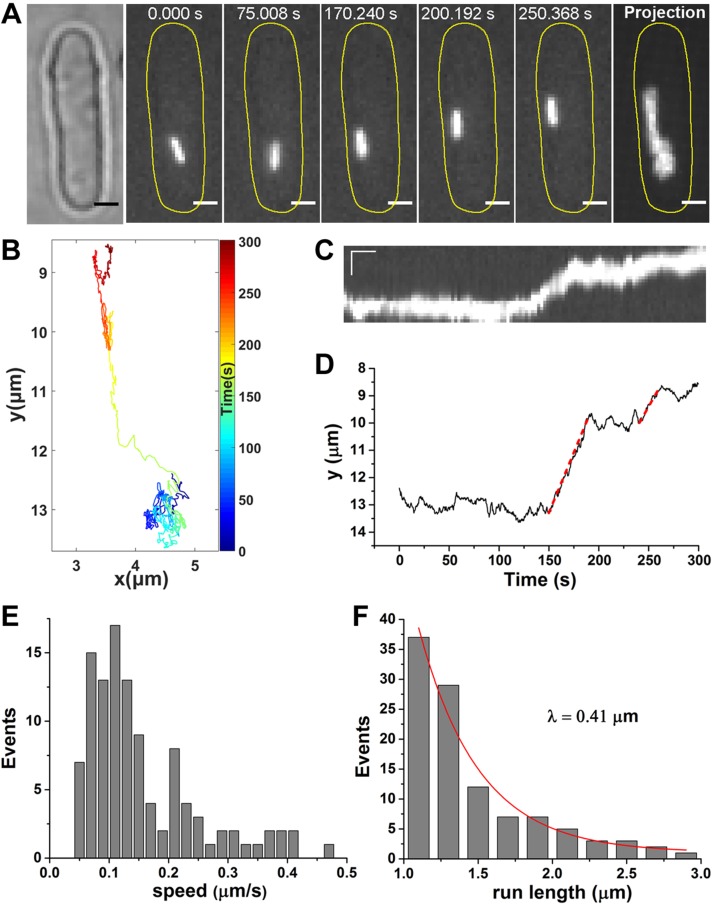
Disrupting microtubules does not halt active transport of cytoophidia in MBC-treated cells. *A*) Serial images of a cytoophidium in an MBC-treated cell show the directed motion. The cells were treated with 25 μg/ml MBC for 30 min to disrupt microtubules before imaging. Far right, the maximum projection of the clip. Scale bars, 2 µm. *B*) Trajectory of the cytoophidium with colored segments corresponding to the time points. *C*) Kymograph of the cytoophidium. Scale bars, 2 µm (vertical) and 20 s (horizontal). *D*) Position of cytoophidium in the MBC-treated cell *vs*. time elapsed, with 2 directed phases marked by red dashed lines. *E*) The speed distribution of the directed phases of cytoophidia. *F*) The run-length distribution of the directed phases of cytoophidia. The red curve is the exponential fit used to determine the run length λ by the same method used in the control experiments. There were 108 directed phases.

In eukaryotic cells, active transport is driven by motor proteins along 2 types of cytoskeleton: microtubules and actin filaments. To further investigate the origin of the active motion of cytoophidia, we examined the dynamic feature of cytoophidia after disrupting these 2 types of cytoskeleton separately. First, prior to imaging, we treated cells with 25 μg/ml MBC for 30 min to disrupt the microtubules, as previously reported (23–25). MBC efficacy was demonstrated by checking the CFP-tagged tubulin strain (Supplemental Fig. S2A). Similar to the control experiment, cytoophidia were still dynamic in the MBC-treated cells (**Fig. 2*A–C*** and Supplemental Movie S2). There were 2 obvious directed phases (red dashed lines) during the imaging (Fig. 2*D*). In the 108 directed phases selected from 129 cytoophidium trajectories, both the speed (0.16 μm/s) and run length (λ = 0.41 μm) in MBC-treated cells were consistent with the control experiments, indicating that microtubules are not involved in the active transport of cytoophidia (Fig. 2*E, F**)*.

### Active transport of cytoophidia occurs along actin filaments

**Figure 3:**
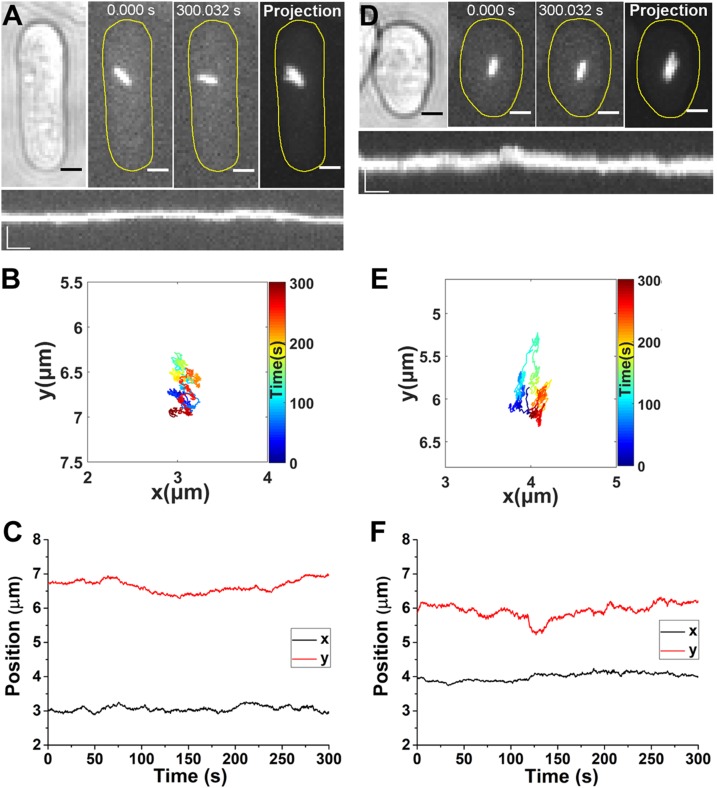
Actin filaments and Myo52 are both indispensable to directed motion of cytoophidia. *A*) Time-lapse images of a cytoophidium in a LatA-treated cell. The cells were treated with 10 µM LatA for 30 min to disrupt the actin filaments before imaging. Far right, the maximum projection of the clip. Scale bars, 2 µm. Lower panel, kymograph of the cytoophidium. Scale bars, 2 µm (vertical) and 20 s (horizontal). *B*) Trajectory of the cytoophidium in the LatA-treated cell, with colored segments corresponding to the time points. *C*) Position of cytoophidium in the LatA-treated cell *vs*. time elapsed. *D*) Time-lapse images of a cytoophidium in a *myo52Δ* cell (in which the gene for Myo52 has been deleted). Far right, the maximum projection of the clip. Scale bars, 2 µm. Lower panel, kymograph of the cytoophidium. Scale bars, 2 µm (vertical) and 20 s (horizontal). *E*) Trajectory of the cytoophidium in the *myo52Δ* cell, with colored segments corresponding to the time points. *F*) *x* and *y* position *vs.* time of the cytoophidium in the *myo52Δ* cell.

Next, we used 10 µM LatA for 30 min to disrupt the actin filaments, as previously reported (23, 24). No actin filaments could be detected after LatA treatment in the *S. pombe* cells expressing Lifeact-GFP (Supplemental Fig. S2B). We show that LatA treatment significantly reduced the movement of an individual cytoophidium (**Fig. 3*A, B*** and Supplemental Movie S3). The cytoophidium was confined to a small area for the entire 5-min imaging period, and there was no obvious directed phase in either the *x*(*t*) or *y*(*t*) plot (Fig. 3*C*). The directed motion was fully eliminated in a total of 75 cytoophidium trajectories after the actin filaments of LatA-treated cells were disrupted.

### Myo52 is a motor protein that transports cytoophidia

Myosins are motor proteins that transport cargo along actin filaments. We next aimed to find out which kind of myosin is in charge of the active transport of cytoophidia. In *S. pombe*, there are 3 major classes of myosin: myosin I, II, and V. Only Myo52, a member of the myosin V class, is known to be involved in the delivery of vesicles and organelles (26, 27). To test whether Myo52 is involved in the directed transport of cytoophidia, we used PCR-based gene targeting to delete the relevant gene. The *myo52Δ* cells showed an altered morphology, being shorter and broader (23, 28). Movement of filamentous cytoophidia in *myo52Δ* cells was suppressed (Fig. 3*D, E* and Supplemental Movie S4), and no eligible directed motion could be detected in the *x*(*t*) and *y*(*t*) plots (Fig. 3*F*). A previous study showed that myosin V can organize the actin cables in fission yeast, and that the depletion of both Myo51 and Myo52 causes an obvious defect in actin cable organization (29). The organization of actin cables in Myo52-depleted *S. pombe* cells, however, is comparable to that of wild-type cells (28, 29). Because we depleted only Myo52 in *S. pombe*, we assumed that the actin cables were not significantly affected. Therefore, our results suggest a direct correlation between Myo52 and the active transport of cytoophidia.

To further test whether Myo52 plays a role in the active transport of cytoophidia, we treated the cells with 2 µM PBP to inhibit Myo52 activity (30). PBP, as a myosin V inhibitor, could mimic the effect of Myo2p mutation in budding *Saccharomyces cerevisiae* (31), another species of yeast, in which Myo2p is both structurally and functionally orthologous to Myo52 in *S. pombe* (23, 28). We found that the movement of cytoophidia was almost completely suppressed after the PBP treatment and no directed phase could be observed (Supplemental Fig. S3). Notably, the actin filaments remained unaffected upon PBP treatment. It has been shown that PBP has effects on other classes of myosin at higher concentrations than that required to inhibit myosin V (31). For example, PBP inhibits ATPase activity of myosin II with 50% inhibitory concentration (IC_50_) at 25 µM, which is 10-fold greater than the concentration we used here (2 µM). Although myosin V is the primary target of PBP, we cannot completely rule out the possibility that other motors also contribute, especially if these motors share a myosin V–dependent anchorage complex. However, the result further demonstrates that Myo52 is involved in the active transport of cytoophidia.

Previous studies reported that the mean speed of Myo52 in *S. pombe* has a wide range of 0.5–2 μm/s (23, 24, 29, 32), faster than that of cytoophidia we measured in control and MBC-treated cells (0.15 μm/s). This is mainly due to the great difference in the size of the objects investigated. In these studies, the Myo52 proteins were labeled and tracked; in our study, however, the much larger cytoophidia were tracked. It is known that the intracellular environment is crowded with molecules and organelles (33, 34), so the speed of the cytoophidia driven by the Myo52 proteins would decrease. The speed of cytoophidia (0.15–0.4 μm/s) is consistent with the myosin V–dependent movement of vacuoles (35) and peroxisomes (36) in budding yeast.

### Statistical analysis further confirms the finding

**Figure 4:**
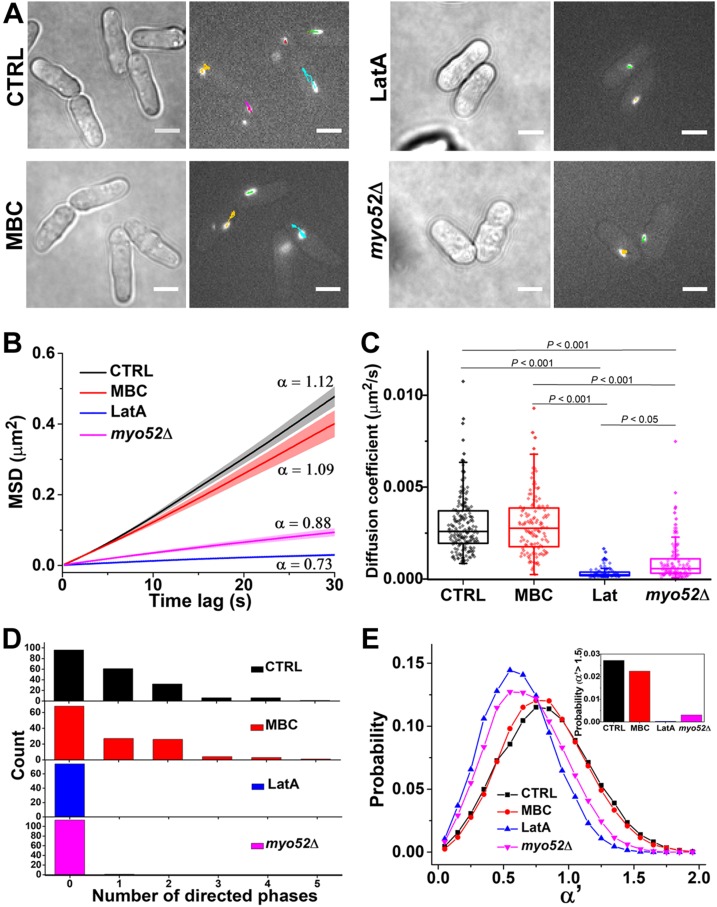
Comparison of cytoophidium dynamics in control, MBC-treated, LatA-treated, and *myo52Δ* cells. *A*) Representative bright-field and fluorescence micrographs show distinct dynamic behaviors under the different conditions. In the fluorescence images, the trajectory of each cytoophidium during 5 min imaging is marked by colored lines. Scale bars, 5 µm. *B*) Plot of average MSD curves as a function of time lag. Error bars represent se. Each MSD curve is calculated from 1 cytoophidium trajectory. Trajectories analyzed: control (*n* = 202), MBC (*n* = 129), LatA (*n* = 75), *myo52Δ* (*n* = 114). *C*) Comparison of diffusion coefficients of cytoophidia under each condition. Each diffusion coefficient was determined by linear fitting of the MSD curve for each trajectory. Diamonds represent samples of diffusion coefficients. Box plots show median and interquartile range. All significant differences are indicated by *P* values, which were calculated using 1-way ANOVA and Tukey’s *post hoc* test. Values of *P* < 0.05 were considered statistically significant. *D*) Distribution of the directed phases from each trajectory over the course of 5 min imaging. Directed phases: control (175), MBC (108), LatA (0), *myo52Δ* (1). *E*) Distributions of temporal exponent α′ of local trajectories (segments of 40 consecutive points along all the cytoophidium trajectories) under different conditions. Inset, the probability of α′ > 1.5: control (0.027), MBC (0.022), LatA (0.0003), *myo52Δ* (0.003). The number of temporal exponents α′ in (*E*): control (199987), MBC (137503), LatA (84658), *myo52Δ* (124285).

The statistical analysis was applied to trajectories of cytoophidia in control (*n* = 202), MBC-treated (*n* = 129), LatA-treated (*n* = 75), and *myo52Δ* (*n* = 114) cells, further supporting our findings (**Fig. 4*A***). In Fig. 4*B*, the mean square displacement (MSD) plots for the 4 groups show significant differences. The MSD plots for the control and MBC-treated cells trend slightly upward, indicating that the cytoophidia under these 2 conditions underwent active transport. For the LatA-treated and *myo52Δ* cells, however, α < 1, indicating that the cytoophidia were subdiffusive and the active transport was abolished under these 2 conditions. The diffusion coefficients for the cytoophidia in control (0.0029 ± 0.0016 μm^2^/s) and MBC-treated cells (0.0030 ± 0.0016 μm^2^/s) are almost the same, and larger than those for the LatA-treated cells (0.00035 ± 0.0003 μm^2^/s) and *myo52Δ* cells (0.00095 ± 0.0011 μm^2^/s) (Fig. 4*C*). Note that the diffusion of cytoophidia in *myo52Δ* cells is slightly faster than that in LatA-treated cells, as well as the increased α of the mean MSD plot. This is due to the random fluctuations of actin filaments that contribute to the intracellular diffusion, in accordance with recent studies (37, 38). Because the diffusion rate is also affected by molecule size, we compared the fluorescence intensities of cytoophidia, which are proportional to the size of cytoophidia (Supplemental Fig. S4). The data shows that, excluding the intensity of the MBC-treated group (∼10% larger than the others), the intensities of the LatA-treated and *myo52Δ* groups were both similar to the control group, eliminating the influence of cytoophidium size on the decreased cytoophidia diffusion in LatA-treated and *myo52Δ* cells.

Next, we compared the distribution of the number of directed phases in each trajectory during the 5-min imaging period (Fig. 4*D*). The elimination of directed phases in the LatA-treated and *myo52Δ* cells clearly demonstrates that the active transport of cytoophidia is driven by Myo52 along the actin filaments. The mean number of directed phases in each trajectory was 0.85 and 0.83 in control and MBC-treated cells, respectively, whereas there were 0 and 0.009 directed phases in LatA-treated and *myo52Δ* cells, respectively. We noticed that although not every trajectory contains a directed phase in control cells, there is still a high occurrence rate of active transport of cytoophidia when compared with the active transport of endocytic vesicles in mammalian cells in which <50% of the trajectories contain the directed segments (39). Note that although the random fluctuation of actin filaments in *myo52Δ* cells may occasionally cause directed movements (only 1 directed phase out of 114 trajectories), only Myo52 could consistently drive the cytoophidia, providing the power for directed motion.

To further determine whether our method was correct and sufficient to detect the directed motion, a local MSD window algorithm was applied to obtain the temporal dynamic behavior along a trajectory (40): MSD_local_(τ) ∼ τ^α′^, where α′ indicates the motion mode of local trajectories (*i.e.*, segments composed of 40 consecutive points on a given trajectory), in contrast to the MSD analysis of whole trajectories. Because there are only a limited number of points to fit along local trajectories, the temporal α′ has a broader fluctuation. In our previous study (40), we showed that an α′ value within the range 0–1.5 corresponds to confined motion and free diffusion. The existence of directed movement driven by motor proteins, then, is indicated when α′ > 1.5. Of all the cytoophidia trajectories in the 4 groups that we studied, we noted that α′ > 1.5 in control and MBC-treated cells but not in LatA-treated and *myo52Δ* cells, indicating the existence of active transport of cytoophidia in the former 2 groups and the suppression of active transport in the latter 2 groups (Fig. 4*E*).

## DISCUSSION

The detection of active transport of cytoophidia raises many interesting questions. For example, when and where does active transport of cytoophidia occur and why do cytoophidia need to be actively transported? Spatially defined organelle activity occurs in many cell types, especially in polarized or spatially extended cells such as epithelial cells or neurons (41). Increasing evidence demonstrates that organelle positioning correlates with function. Local organelle positioning requires active transport followed by immobilization. Recently, several studies have shown that filamentation of CTPS serves to sequester or strengthen enzymatic activity in response to environmental and developmental stresses (42–45). Therefore, it is conceivable that local positioning of cytoophidia *via* active transport is important for metabolic regulation.

In our previous studies, we found that cytoophidia are motile but not actively transported in *Saccharomyces cerevisiae* (46, 47). The assembly of cytoophidia seems different between budding yeast and fission yeast because cytoophidia appear at the log phase and disappear at the stationary phase in *S. pombe*. By contrast, cytoophidia appear in the stationary phase but disappear at the log phase in *S. cerevisiae*. So, a possible reason that cytoophidia weren’t actively transported could be that the budding yeast lacked sufficient energy in the stationary phase. Considering fission yeast and budding yeast are divergent species and separated in the evolutionary tree around 400 million years ago, cytoophidia may be regulated in different ways in each species. Many additional proteins form independent cytoophidia in budding yeast, although the existence and movement of similar cytoophidia in fission yeast have yet to be determined. Further studies are required to understand the differences in cytoophidium assembly and regulation between fission yeast and budding yeast.

Myosin V motors progressively transport a variety of cargo along actin filaments (48, 49). In vertebrates, myosin V proteins transport melanosomes in melanocytes and endoplasmic reticuli in neurons. In budding yeast, myosin V motors transport membrane-bound organelles as well as mRNA from the mother cell to the bud. Thus, our finding that myosin V also recognizes membraneless organelles such as cytoophidia is significant. In addition to CTPS, several other metabolic enzymes such as inosine-5′-monophosphate dehydrogenase and glutamine synthase can form cytoophidium-like filamentous structures (14, 50–52). Further studies are required to identify the adaptor proteins that assist the binding of myosin V and cytoophidia.

Through single-particle tracking of CTPS–containing cytoophidia in individual live cells, we have shown that motor proteins drive the active transport of cytoophidia in *S. pombe*. More importantly, our results indicate that actin filaments, not microtubules, mediate the active transport of cytoophidia. In addition, we have demonstrated that myosin V plays critical roles in the active transport of cytoophidia. The compartmentation of metabolic enzymes such as CTPS and inosine-5′-monophosphate dehydrogenase into the cytoophidium and its kind provides an excellent model to study the cellular mechanisms responsible for metabolic regulation *via* filamentation. Because the cytoophidium represents a novel type of membraneless organelle, our results are an important step toward understanding how the behavior and dynamics of these intracellular compartments contribute to the local regulation of metabolism.

## Supplementary Material

This article includes supplemental data. Please visit *http://www.fasebj.org* to obtain this information.

## Abbreviations

CTPS: cytidine triphosphate synthase
LatA: latrunculin A
MBC: carbendazim
MSD: mean square displacement
PBP: pentabromopseudilin
YFP: yellow fluorescent protein
