# Supplementary material for: Active transport of cytoophidia in Schizosaccharomyces pombe
Source: FASEB J. 2018 May 21;32(11):5891–8. doi: 10.1096/fj.201800045RR (PMC6292696; doi:10.1096/fj.201800045RR)
Supplement: Supplementary file 5 [file fj.201800045RR.sd1.docx]

**Supplemental Materials**

**
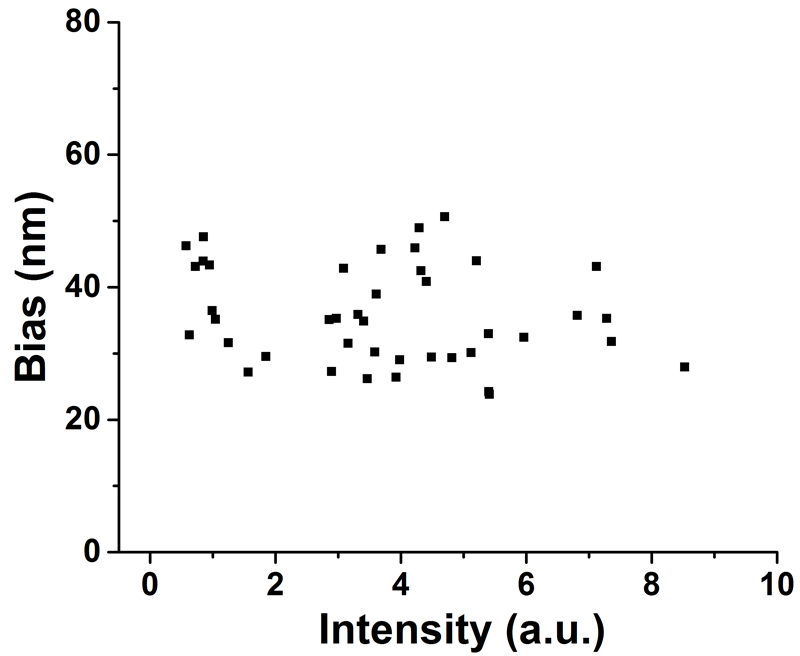
**

**Figure S1.** The location accuracy for the cytoophidia is independent of their lengths. The cells were fixed in 2% PFA for 10min and imaged under the same conditions as the live cells. Note that the prolonged PFA fixation may affect the fluorescence signal. Each dot represents a standard deviation of multiple localization measurements of a cytoophidium. The fluorescence intensities of cytoophidia correspond to their lengths. The mean bias is 35.8 nm, both for the short and long cytoophidia (n = 42).


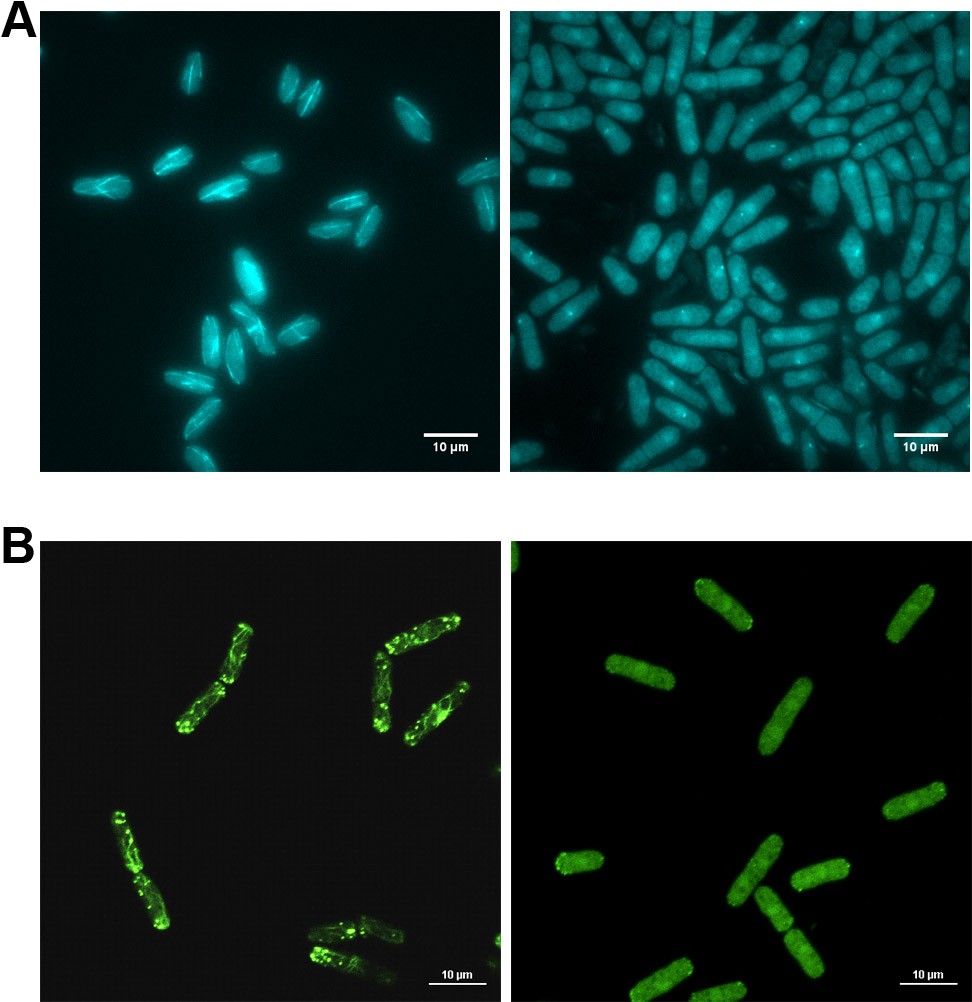


**Figure S2. Microtubules and actin filaments are disrupted respectively.** *A*) 25 µg/ml MBC is effective in disrupting microtubules in *S. pombe* cells expressing tubulin-CFP. Left, control cells; right, treated cells. *B*) 10 µM LatA is effective in disrupting actin filaments in *S. pombe* cells expressing Lifeact-GFP. Left, control cells; right, treated cells. Cells were treated by drugs for 30min prior to the imaging. Scale bar, 10 µm.


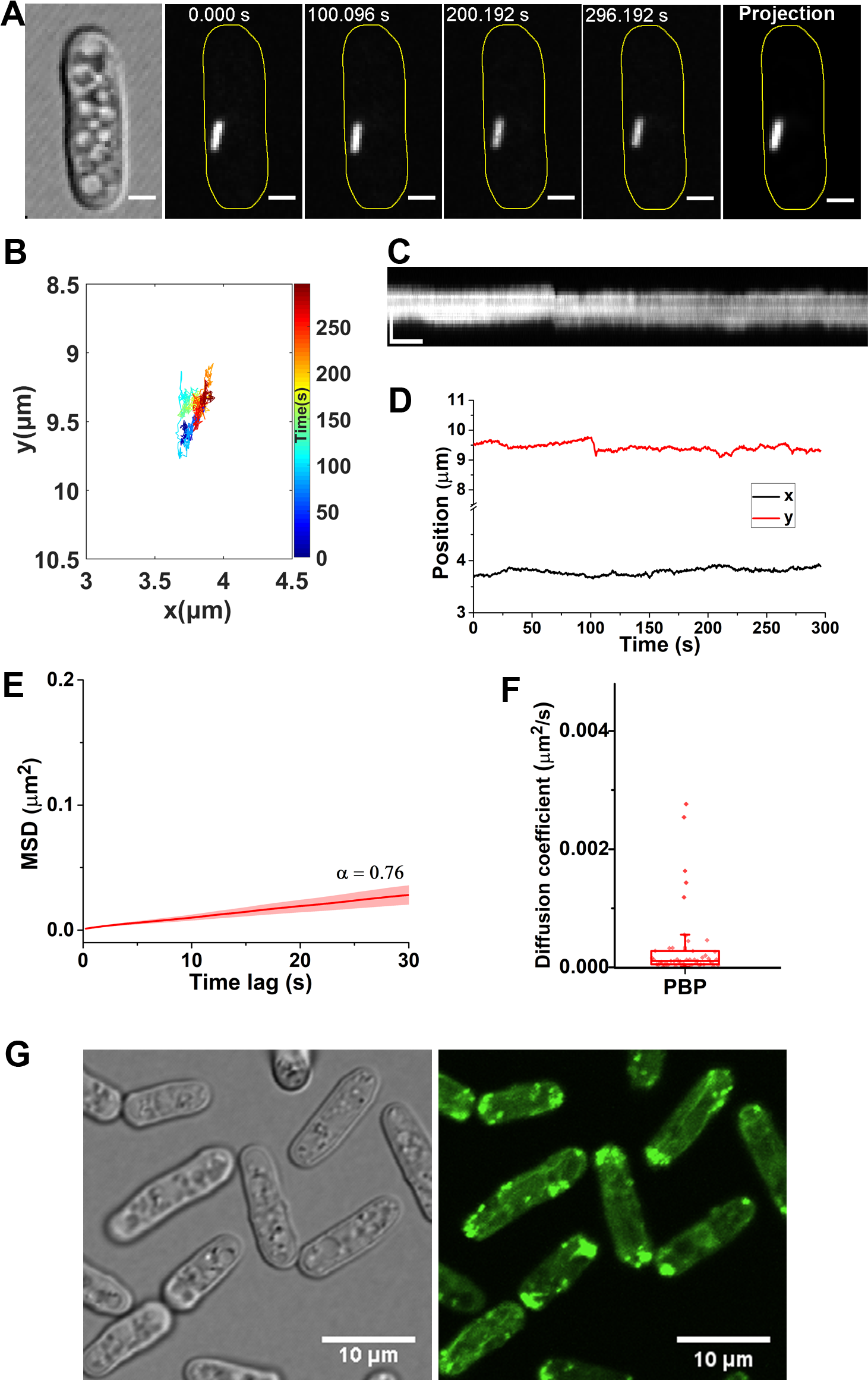


**Figure S3.** Active transport of cytoophidia is abolished in PBP-treated cells. *A*) Time-lapse images of a cytoophidium in a PBP-treated cell. The cell were treated with 2 µM PBP for 30 min to inhibit Myo52 activity before imaging. Far right, maximum projection of the movie. Scale bar is 2 µm. *B*) Trajectory of the cytoophidium in the PBP-treated cell, with colored segments corresponding to the time points. *C*) Kymograph of the cytoophidium. Scale bar, 2 µm (vertical) and 20s (horizontal). *D*) x and y position versus time of the cytoophidium in the PBP-treated cell. *E*) Average MSD curve as a function of time lag. Error bars represent standard error. The sample number of trajectories for analysis is 50. *F*) Diffusion coefficient of cytoophidia in the PBP-treated cells. Each diffusion coefficient is determined by linear fitting of the MSD curve for each trajectory. The mean diffusion coefficients is 0.0003±0.0001 µm^2^/s. Diamonds represent samples of diffusion coefficients. Box plots show median and IQR. *G*) The actin filaments remain unaffected in the PBP-treated cells. Left, bright field; right, Lifeact-GFP.


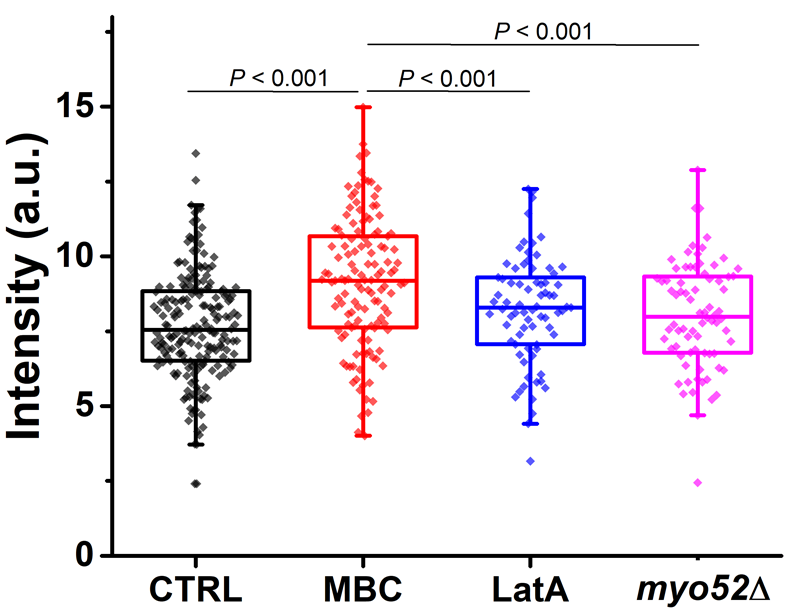


**Figure S4.** Fluorescence intensities of cytoophidia in different conditions. The number of cytoophidia for analysis: control (n = 202), MBC (n = 129), LatA (n = 75), *myo52Δ* (n = 114). Diamonds represent intensity samples. Box plots show median and IQR. All significant differences are indicated by *P* values, which were calculated by one-way ANOVA and post hoc Tukey’s test. *P* < 0.05 was considered to be statistically significant. The intensities for control, LatA-treated and *myo52Δ* groups have no significant differences, indicating the cytoophidia are in the same size in these three groups. In MBC-treated cells, the cytoophidium intensity is significant different from the other groups, but the average intensity is only about 10% larger, probably because the cytoophidium size slightly increases in the longer *S. pombes* due to the MBC treatment.

**Supplemental Movie Legends**

**Movie S1.** A cytoophidium undergoes active movement in the *S. pombe* cell. CTP synthase was fused to YFP and expressed under endogenous promoter. *S. pombe* cells were treated with 1% DMSO as the control group. As in all the following movies, the original images were acquired every 0.2558s for 5 min. The number of frames is reduced for smaller file size and the movie is played at 10 frames/s.

**Movie S2.** A cytoophidium undergoes active movement in the MBC-treated cell. The *S. pombe* cells were treated with 25 μg/ml MBC for 30 min to disrupt microtubules prior to imaging.

**Movie S3.** A cytoophidium shows motionless behavior in the LatA-treated cell. The *S.pombe* cells were treated with 10 µM LatA for 30 min to disrupt the actin filaments before imaging.

**Movie S4.** A cytoophidium shows motionless behavior in the *myo52△* cell. The gene for Myo52 has been deleted in the cell.
